# Nurses’ knowledge and attitudes toward patient-controlled analgesia for postoperative pain control in a tertiary hospital in South Korea

**DOI:** 10.1186/s12912-022-01106-7

**Published:** 2022-11-22

**Authors:** Mi-Ra Kang, Youn-Ju Kwon

**Affiliations:** 1 Department of Nursing, Acute Pain Service Team, Asan Medical Center, 88, Olympic-ro 43-gil, Songpa-gu, Seoul, South Korea; 2 Unit Manager, Department of Nursing, Post Anesthetic Care Unit, Asan Medical Center, Seoul, South Korea

**Keywords:** Nurse, Patient-controlled analgesia, Postoperative pain, Knowledge, Attitudes

## Abstract

**Background:**

This study investigated the knowledge and attitude of surgical ward nurses toward patient-controlled analgesia (PCA) to develop educational material for nurses on the use of PCA.

**Methods:**

This study was a cross-sectional study comprising 120 nurses from eight surgical wards in a tertiary hospital in South Korea. A questionnaire addressing 6 domains of knowledge of and attitudes towards PCA was conducted over 1 week and analyzed using descriptive and inferential statistical methods. Knowledge was measured on a categorical scale of 0 and 1 (20 points), and attitude was measured on a Likert scale of 1 to 4 points (60 points).

**Results:**

The total score quantifying the knowledge of and attitudes toward PCA of surgical ward nurses was 59.5 ± 5.5 out of 80.0 points. The average age of the subjects was 28.58 ± 5.68 years old, and nurses above the age of 28 had significantly greater knowledge and better attitudes (61.7 ± 5.5) than those below the age of 28 (57.9 ± 4.9) (*p* < .001). Nurses working on the upper abdominal surgical ward had significantly greater knowledge (16.2 ± 1.9) than nurses working on other wards (thorax: 14.0 ± 2.3, lower abdominal: 15.4 ± 1.9, and musculoskeletal: 14.5 ± 2.2) (*p* = .001). Nurses who received education about PCA had significantly better attitudes (45.3 ± 4.6) than those who did not (41.3 ± 3.5) (*p* < .001). The average correct answer rate for knowledge of opioid analgesics was lower (68.2%) than that for knowledge of the basic configuration of PCA equipment (73.3%) and areas to be identified and managed when using PCA (84.6%), and there was a significant correlation with attitudes toward side effect management (*p* < .05, *r* = .19).

**Conclusions:**

There was a significant correlation between the knowledge and attitude of nurses regarding opioid use in PCA. Older nurses with greater clinical experience on the surgical wards who had received PCA education had a better attitude toward PCA. Therefore, newly trained nurses on surgical wards with no experience of PCA education should undergo an intensive education program on opioid analgesics used in PCA.

## Background

Postoperative pain is one of the greatest concerns for patients preparing for surgery and has considerable effect on patient satisfaction after surgery [1]. Inadequate postoperative pain control results in various complications such as hypertension, tachycardia, atelectasis, gastrointestinal obstruction, delayed wound healing, and chronic pain [2, 3]. Patient-controlled analgesia (PCA) is used worldwide as a method to control postoperative pain due to its effectiveness, safety, and cost-effectiveness and the associated increase in patient satisfaction [4–10].

Despite technological advancements and pharmaceutical developments, postoperative pain control remains generally inadequate, and evidence indicates that the knowledge and attitudes of nurses regarding opioid analgesics have an important effect on pain control [3, 11, 12]. Studies have reported that patients showed a passive attitude toward pain expression and the use of opioid analgesics and expressed the inappropriate belief that using multiple opioid analgesics would lead to addiction and delay wound recovery [11, 13]. Doctors and nurses were more concerned about addiction to opioid analgesics than patients, expressed strong concerns about the occurrence of respiratory depression, and were also shown to underestimate the pain intensity expressed by patients [3, 12, 13]. Furthermore, a study found that although nausea and vomiting after surgery were not side effects of PCA, PCA was discontinued due to inaccurate knowledge of the surgical nurses [14].

In the tertiary general hospital A where this study was conducted, a nurse-based, anesthesiologist-supervised acute pain service (APS) oversees PCA management; the APS has been in operation for over 10 years. Three advanced practice nurses and two professors from the department of anesthesiology and pain medicine are in charge of postoperative pain management through the APS, and advanced practice nurses mainly educate nurses, patient, and guardians on the surgical ward. Institution A provides pain management education programs through a variety of methods. New nurses entering the institution for the first time are required to participate in pain management education programs and are encouraged to freely participate in education programs thereafter. The PCA education program is included in the pain management education program. Advanced practice APS nurses also educate surgical nurses on an individual basis, responding to questions about PCA or postoperative pain management methods.

Since patients are influenced by the education and explanations they receive—from pain assessment to intervention—nurses play the most important role in pain management [12]. Recently, the involvement of nurses in pain management has attracted considerable research interest; however, most studies have focused on the overall knowledge of nurses and their attitude toward pain management [12, 13, 15–19]. The effect of nurse education on the management of PCA has also been investigated [8, 14, 20–22]. However, few studies in Korea or internationally have investigated the knowledge and attitudes of nurses responsible for the delivery of information on PCA for postoperative pain. In addition, the nurses’ knowledge about the use of PCA drugs in the studies was inadequate [23–25]. The level of knowledge of nurses about PCA and their attitude toward its use affects pain control in patients requiring PCA. Therefore, it is important to identify deficiencies in nurses’ knowledge about PCA used for postoperative pain control, identify vulnerable areas, and implement training programs addressing these issues to optimize the use of PCA for effective postoperative pain control.

## Purpose

This study was conducted to confirm the knowledge and attitude of surgical nurses toward PCA and use this information to develop future nurse educational materials regarding PCA use. Specifically, we aimed to:

1) Assess nurses’ knowledge and attitudes toward PCA.

2) Identify factors that influence nurses’ knowledge and attitudes toward PCA.

## Methods

### Design and sample

This was a cross-sectional study of 120 nurses working in surgical wards. This study was approved by Institutional Review Board of Asan Medical Center in Seoul, South Korea (IRB No: 2021-1372). Cluster sampling was used to recruit eight surgical wards (2 thoracic surgical wards, 2 upper abdominal surgical wards, 2 lower abdominal surgical wards, and 2 musculoskeletal surgical wards) with the highest postoperative PCA usage among the 22 surgical wards of Asan Medical Center. The participants were nurses who had worked for over 3 months on the surgical ward and were sufficiently exposed to the use of PCA. New nurses who worked on the surgical ward for more than 3 months were also included in the study because provide direct education to patients on how to use PCA or manage its side effects. The minimum sample size of 109 participants was calculated using G*Power 3.1.9.7 for linear multiple regression (fixed model, R^2^ deviation from zero) based on an effect size of 0.15, a significance level of 0.05, a power of 0.80, and 8 predictors; therefore, a total of 120 participants was obtained in consideration of a dropout rate of 10%. A total of 119 questionnaires were retrieved, with a recovery rate of 99.1%. Among the collected questionnaires, there were two copies with omitted answers (1.7%), and 117 final questionnaires were included in the analysis (98.3%).

### Instruments

The tool used to confirm the knowledge and attitudes of surgical ward nurses toward PCA was a questionnaire tool developed by the researchers based on a literature review [3–6, 8, 10–13, 16–19, 22, 23, 25–27]. Existing tools were not used because there is limited research on nurses’ knowledge and attitudes toward PCA. Furthermore, previous studies only confirmed the nurses’ knowledge of PCA or focused on newly developed types of PCA [23, 25]. Six experts verified the validity of the questionnaire tool used in this study. The scale’s total content validity universal agreement (S-CVI/UA) index was 0.88, and Cronbach’s α was 0.68. In detail, the Kuder–Richardson Formula 20 value of the internal consistency of knowledge, which was a dichotomy variable, was 0.64 (items 1–4, *r* = 0.63; items 5–13, *r* = 0.57; and items 14–20, *r* = 0.72). Cronbach’s α of internal consistency of the attitude was 0.72 (items 21–27, *r* = 0.63; items 28–32 *r* = 0.70; and items 33–35, *r* = 0.84).

The questionnaire was written and developed in Korean, and the nurses independently read the questions and filled out the answers. Before this survey, a preliminary survey involving five surgical ward nurses was conducted to confirm whether the survey tool was appropriate for nurses and whether it was easy to understand. The five nurses included in the preliminary survey were not included in the final analysis.

The questionnaire was divided into three areas of knowledge and three areas of attitude and comprised a total of 35 questions. The tool included 20 items with a “Yes/No” response and 15 items with a four-point Likert scale response (strongly disagree, disagree, agree, and strongly agree). For “Yes/No” responses, incorrect answers received 0.0 points and correct answers received 1.0 points; on the four-point Likert scale, the most inappropriate answer received 1.0 points and the most appropriate answer received 4.0 points. Therefore, the survey was scored from a minimum of 15.0 points to a maximum of 80.0 points. Higher questionnaire scores indicate higher levels of knowledge and more appropriate attitudes.

### Data analysis

Descriptive statistics, t-test, one-way analysis of variance (ANOVA), Pearson correlation coefficient, and multiple linear regressions were performed using SPSS Statistics 21.0 for Windows (IBM Inc., Armonk, NY, USA), and the Scheffé test was used for post-analysis after one-way ANOVA. Two-tailed statistical significance was set at a *p*-value of .05. Descriptive statistics using percentages, means, and standard deviations were used to determine the general characteristics of the participants, knowledge, and attitude scores. The t-test was used to analyze the nurses’ knowledge and attitudes according to sex, age, educational degree, and experience of receiving education on PCA. One-way ANOVA and Scheffé test were used to analyze nurses’ knowledge and attitudes according to clinical experience, clinical surgical ward experience, and surgical ward type. The Pearson correlation coefficient was used to analyze the correlation between the subdomain areas of knowledge and attitude toward PCA. Multiple linear regression was used to analyze factors influencing nurses’ knowledge and attitudes toward PCA.

## Results

### Knowledge and attitude toward PCA according to the general characteristics of nurses

The scores for total knowledge and attitude of surgical ward nurses toward PCA were 59.5 ± 5.5 out of 80.0 points, 74.3 points in percentile conversion. Overall, 92.3% of the participants were women, and 56.4% of the nurses had 1-5 years of clinical experience in surgical wards. Additionally, 91.5% had general pain management education experience, and 79.5% had PCA education experience. The participants’ average age was 28.58 ± 5.68 years. This average age (28 years) was used as a cutoff in the analysis to identify differences between the group under 28 years of age and that over 28 years of age. Based on this parameter, we found that nurses above the age of 28 (61.7 ± 5.5) had significantly higher knowledge and better attitude scores for PCA than those below the age of 28 (57.9 ± 4.9; *p* < .001). The group with 1 to 5 years of clinical experience of the surgical ward had significantly lower scores for knowledge and attitude (57.7 ± 5.0) than the group with 5 to 10 years (62.2 ± 6.2) and the group with over 10 years’ experience (62.6 ± 5.3) (*p* < .001) (Table 1).

**Table 1:** PCA knowledge and attitudes according to the general characteristics of surgical ward nurses

Characteristics	*N* (%) or Mean (SD)	Knowledge		Attitudes		Total	
		Mean (SD)	t or F (*p*)	Mean (SD)	t or F (*p*)	Mean (SD)	t or F (*p*)
Total	117 (100.0)	15.0 (2.2)		44.4 (4.7)		59.5 (5.5)	
Sex							
F	108 (92.3)	15.0 (2.2)	t = −0.34 (.734)	44.4 (4.7)	t = − 0.15 (.885)	59.4 (5.5)	t = − 0.26 (.796)
M	9 (7.7)	15.2 (2.4)		44.7 (4.0)		59.9 (5.6)	
Age (yr)	28.58 (5.68)						
<28	69 (59.0)	14.6 (2.3)	t = −2.48 (.014)	43.3 (4.1)	t = −3.33 (.001)	57.9 (4.9)	t = −3.92 (<.001)
≥28	48 (41.0)	15.6 (2.0)		46.1 (4.9)		61.7 (5.5)	
Clinical experience (yr)	2.46 (0.92)						
<1^a^	12 (10.3)	13.9 (2.0)	F = 2.55 (.059)	45.4 (3.7)	F = 8.18 (<.001) c, d > b†	59.3 (3.7)	F = 8.49 (<.001) c, d > b†
1–<5^b^	62 (53.0)	14.8 (2.4)		42.7 (4.1)		57.5 (5.0)	
5–<10^c^	20 (17.1)	15.6 (1.9)		46.1 (5.3)		61.7 (6.0)	
≥10^d^	23 (19.7)	15.7 (1.9)		47.3 ( 4.1)		63.0 (4.8)	
Clinical surgical ward experience (yr)	2.37 (0.88)						
< 1^a^	13 (11.1)	14.4 (2.5)	F = 2.71 (.049) c, d > b	45.2 (3.6)	F = 5.20 (.002) c, d > b†	59.6 (3.7)	F = 6.66 (<.001) c, d > b†
1–<5^b^	66 (56.4)	14.7 (2.2)		43.1 (4.2)		57.7 (5.0)	
5–<10^c^	20 (17.1)	15.8 (1.9)		46.4 (5.4)		62.2 (6.2)	
≥10^d^	18 (15.4)	15.9 (1.9)		46.7 (4.4)		62.6 (5.3)	
Education							
Bachelor	104 (88.9)	14.9 (2.2)	t = −1.55 (.124)	44.1 (4.6)	t = −2.34 (.021)	59.0 (5.4)	t = −2.64 (.009)
≥Postgraduate	13 (11.1)	15.9 (2.2)		47.2 (3.9)		63.2 (4.6)	
Experience of education on pain management							
Yes	107 (91.5)	15.0 (2.2)	t = −0.19 (.853)	44.7 (4.7)	t = −2.19 (.030)	59.8 (5.5)	t = −1.93 (.056)
No	10 (8.5)	14.9 (2.3)		41.4 (3.3)		56.3 (3.7)	
Experience of education on PCA							
Yes	93 (79.5)	15.1 (2.1)	t = −0.37 (.712)	45.3 (4.6)	t = −4.00 (<.001)	60.3 (5.4)	t = −3.50 (.001)
No	24 (20.5)	14.9 (2.6)		41.3 (3.5)		56.1 (4.5)	
Wards							
Thorax^a^	30 (25.6)	14.0 (2.3)	F = 6.06 (.001) b > a, d†	44.2 (4.5)	F = 1.72 (.166)	58.3 (5.6)	F = 4.02 (.009) b > d†
Upper abdomen^b^	29 (24.8)	16.2 (1.9)		45.9 (4.0)		62.1 (4.3)	
Lower abdomen^c^	30 (25.6)	15.4 (1.9)		44.4 (5.4)		59.8 (5.8)	
Musculoskeletal^d^	28 (23.9)	14.5 (2.2)		43.1 (4.5)		57.6 (5.3)	

(*N* = 117)

*M* male; *F* female; *SD* standard deviation; *PCA* patient-controlled analgesia; † Scheffé test; Thorax = heart and lung surgery; Upper abdomen = liver and kidney surgery; Lower abdomen = colorectal and gynecological surgery; Musculoskelatal = limbs and spine surgery

### Nurses’ knowledge of PCA

In this study, the average correct answer rate for opioid analgesics (68.2%) was lower than that for the basic configuration of PCA equipment (73.3%) and areas to be identified and managed when using PCA (84.6%). Nurses who were older with considerable clinical surgical ward experience, worked in the upper abdominal surgical ward had significantly higher knowledge scores than other groups (Table 1). As shown in Table 2, the top 5 items with the highest correct answer rates were as follows: “Patients using PCA should be assessed for pain regularly” (100.0%), “Nurses should check regularly for side effects while using PCA” (100.0%), “The normal operation of the PCA, the amount of injection, and the injection site should be checked regularly” (100.0%), “Postoperative wound healing is delayed due to the use of PCA” (100.0%), and “The most common side effect of PCA use is nausea and vomiting” (97.4%). In general, the correct answer rate was high for items related to management (Table 2).

**Table 2:** Knowledge on PCA of surgical ward nurses

Items	Correct *N* (%)	Incorrect *N* (%)
Basic configuration of PCA equipment		
1. PCA is used only for intravenous injection. (No)	93 (79.5)	24 (20.5)
2. PCA always has on automatically infused dose. (No)	78 (66.7)	39 (33.3)
3. Bolus is used when the pain is unbearably severe. (No)	82 (70.1)	35 (29.9)
4. The PCA lockout interval is the time interval from one use of the bolus to the next available use, all of which are 15 min. (No)	90 (76.9)	27 (23.1)
	86 (73.3) ^a^	31 (26.7) ^a^
Opioid analgesics		
5. PCA includes all opioid analgesics. (No)	47 (40.2)	70 (59.8)
6. PCA is more effective for postoperative pain control than PRN opioid analgesics. (Yes)	65 (55.6)	52 (44.4)
7. Fentanyl citrate is suitable as a PCA drug for patients with impaired renal function. (Yes)	62 (53.0)	55 (47.0)
8. The most common side effect of PCA use is nausea and vomiting. (Yes)	114 (97.4)	3 (2.6)
9. Postoperative wound healing is delayed due to the use of PCA. (No)	117 (100.0)	0 (0.0)
10. The incidence of addiction due to the use of PCA is very rare. (Yes)	100 (85.5)	17 (14.5)
11. The incidence of respiratory depression due to the use of PCA is very rare. (Yes)	86 (73.5)	31 (26.5)
12. Urinary retention is common with PCA use. (No)	88 (75.2)	29 (24.8)
13. If delirium occurs, PCA use should be stopped. (No)	39 (33.3)	78 (66.7)
	79 (68.2)^b^	38 (31.8)^b^
Confirmation and management while using PCA		
14. Consciousness and respiratory depression may occur during PCA use; therefore, the level of consciousness and vital signs should be checked regularly. (Yes)	112 (95.7)	5 (4.3)
15. There is a risk of respiratory depression when using PCA in patients with sleep apnea, obesity, liver, kidney, cardiopulmonary dysfunction, and taking sedatives. (Yes)	110 (94.0)	7 (6.0)
16. Patients using PCA should be assessed for pain regularly. (Yes)	117 (100.0)	0 (0.0)
17. Nurse should check regularly for side effects while using PCA. (Yes)	117 (100.0)	0 (0.0)
18. The normal operation of the PCA, the amount of injection, and the injection site should be checked regularly. (Yes)	117 (100.0)	0 (0.0)
19. The catheter of epidural PCA can be removed at any time, even if bemiparin sodium or enoxaparin sodium is administered. (No)	88 (75.2)	29 (24.8)
20. Education on how to use PCA is effective after surgery in the recovery room or hospital room. (No)	32 (27.4)	84 (71.8)
	99 (84.6)^c^	18 (15.4) ^c^

(*N* = 117)

*PCA* patient-controlled analgesia; *PRN* pro re nata; ^a^ = average number and % of correct/incorrect answers for items 1 to 4; ^b^ = average number and % of correct/incorrect answers for items 5 to 13; ^c^ = average number and % of correct/incorrect answers for items 14 to 20

Conversely, the top 5 items with the lowest correct answer rates were as follows: “Education on how to use PCA is effective after surgery in the recovery room or hospital room” (27.4%), “If delirium occurs, PCA use should be stopped” (33.3%), “PCA includes all opioid analgesics” (40.2%), “Fentanyl citrate is suitable as a PCA drug for patients with impaired renal function” (53.0%), and “PCA is more effective for postoperative pain control than PRN opioid analgesics” (55.6%). In general, the incorrect answer rate was high for knowledge related to opioid analgesics used in PCA (Table 2).

### Nurses’ attitudes toward PCA

Nurses who were older, with considerable clinical surgical ward experience, an educational background above graduate school, and experience in PCA education, had significantly better attitudes toward PCA (Table 1). As shown in Table 3, the top 3 items with the best attitude were as follows: 100.0% of nurses agreed or strongly agreed with “I administer PRN opioid analgesics to patients if their pain control is insufficient even during PCA use,” 99.1% of nurses agreed or strongly agreed with “I regularly assess the patient’s pain using the pain assessment tool each time,” and 98.2% of nurses agreed or strongly agreed with “I regularly check whether the drug and personal information, insertion site, injection volume, and settings of the PCA that the patient is using operate normally.” Overall, the attitude toward items requiring regular management while using PCA was better.

**Table 3:** Attitudes of surgical ward nurses toward PCA

Items	Strongly disagree *N* (%)	Disagree *N* (%)	Agree *N* (%)	Strongly agree *N* (%)
Education on how to use PCA				
21. I educate the patient and guardian on how to use PCA every time.	0 (0.0)	16 (13.7)	44 (37.6)	57 (48.7)
22. I educate the patient on how to use PCA immediately after surgery.	0 (0.0)	9 (7.7)	41 (35.0)	67 (57.3)
23. I educate the patient on how to use PCA before surgery.	24 (20.5)	47 (40.2)	28 (23.9)	18 (15.4)
24. I always educate patients on how to use bolus by demonstrating it directly.	3 (2.6)	15 (12.8)	40 (34.2)	59 (50.4)
25. I educate patients to use the bolus before moving or doing deep breathing exercises.	6 (5.1)	36 (30.8)	37 (31.6)	38 (32.5)
26. I educate patients not to use too much of the bolus as it can cause side effects such as nausea, vomiting, and dizziness.	12 (10.3)	47 (40.2)	48 (41.0)	10 (8.5)
27. I double check that the patient understands how to use PCA.	0 (0.0)	10 (8.5)	75 (64.1)	32 (27.4)
Side effects management of opioid analgesics				
28. I regularly check whether side effects occur in patients using PCA.	0 (0.0)	6 (5.1)	70 (59.9)	41 (35.0)
29. I stop using PCA as the first intervention method when a patient complains of nausea, vomiting and dizziness.	7 (6.0)	34 (29.0)	45 (38.5)	31 (26.5)
30. I first administer an antiemetic drug before stopping PCA if the patient complains of nausea and vomiting.	9 (7.7)	67 (57.3)	26 (22.2)	15 (12.8)
31. If the patient’s pain is continuously severe, I use PCA with antiemetic drugs even if patient has nausea or vomiting symptoms.	6 (5.1)	58 (49.6)	45 (38.5)	8 (6.8)
32. I stop using PCA and administer PRN opioid analgesics if side effects such as nausea, vomiting, and dizziness occur even if the patient’s pain is continuously severe.	1 (0.9)	50 (42.7)	46 (39.3)	20 (17.1)
Confirmation and management of pain & PCA				
33. I regularly assess the patient’s pain using the pain assessment tool each time.	0 (0.0)	1 (0.9)	51 (43.5)	65 (55.6)
34. I administer PRN opioid analgesics to patients if their pain control is insufficient even during PCA use.	0 (0.0)	0 (0.0)	40 (34.2)	77 (65.8)
35. I regularly check whether the drug and personal information, insertion site, injection volume, and settings of the PCA that the patient is using, operate normally.	1 (0.9)	1 (0.9)	53 (45.2)	62 (53.0)

(*N* = 117)

*PCA* patient-controlled analgesia; *PRN* pro re nata

Conversely, the top 3 items that showed the least appropriate attitudes were as follows: 65.0% of nurses agreed or strongly agreed with “I stop using PCA as the first intervention method when a patient complains of nausea, vomiting, and dizziness,” and 65.0% of nurses disagreed or strongly disagreed with “I first administer an antiemetic drug before stopping PCA if the patient complains of nausea and vomiting.” Next, 60.7% of nurses disagreed or strongly disagreed with “I educate the patient on how to use PCA before surgery,” and 56.4% of nurses agreed or strongly agreed with “I stop using PCA and administer PRN opioid analgesics if side effects such as nausea, vomiting, and dizziness occur, even if the patient’s pain is continuously severe.” There was an inappropriate attitude regarding the management of side effects of opioid analgesia (Table 3).

### Correlation between knowledge and attitude subdomains on PCA

This study attempted to determine details of the nurses’ knowledge and attitudes toward PCA that are related to each other. Two knowledge and three attitude subdomains showed significant correlation with each other. Higher total scores were observed with greater knowledge of the basic configuration of PCA equipment (*r* = .34, *p* < .001) and of opioid analgesics (*r* = .50, *p* < .001). Higher total scores were also associated with better attitudes toward PCA usage education (*r* = .73, *p* < .001), management of the side effects of opioid analgesics (*r* = .55, *p* < .001), and confirmation and management of pain and PCA (*r* = .61, *p* < .001). Furthermore, greater knowledge of opioid analgesics was associated with better attitudes toward the management of opioid side effects (*r* = .19, *p* < .05) (Table 4).

**Table 4:** Correlation between knowledge and attitudes subdomains of PCA

Variables	1	2	3	4	5	6
	*r* (*p*)	*r* (*p*)	*r* (*p*)	*r* (*p*)	*r* (*p*)	*r* (*p*)
1. Total	–					
2. (K) Basic configuration of PCA equipment	.34 (<.001)	–				
3. (K) Opioid analgesics	.50 (<.001)	.20 (.033)	–			
4. (K) Confirmation and management while using PCA	.12 (.211)	−.00 (.976)	−.00 (.922)	–		
5. (A) Education on how to use PCA	.73 (<.001)	.11 (.243)	.17 (.067)	−.05 (.582)	–	
6. (A) Side effects management of opioid analgesics	.55 (<.001)	−.11 (.237)	.19 (.042)	−.01 (.930)	.08 (.400)	–
7. (A) Confirmation and management of pain and PCA	.61 (<.001)	.23 (.011)	.06 (.521)	−.03 (.784)	.50 (<.001)	.10 (.318)

(*N* = 117)

*K* knowledge; *A* attitudes; *PCA* patient-controlled analgesia

### Factors affecting nurses’ knowledge and attitudes toward PCA

The degree of education, the experience of receiving education on PCA, clinical experience on the surgical ward, and type of surgical ward were used to identify the variables that most significantly influenced nurses’ knowledge and attitudes toward PCA. The Durbin-Watson statistic for multiple linear regression analysis was 2.08, indicating that there was no autocorrelation. The tolerance between independent variables was 0.64 to 0.95, all of which were 0.10 or higher, and the Variation Inflation Factor was 1.05 to 1.55, indicating no multicollinearity. The results of this study showed that nurses who work in upper abdominal surgery (*β* = .30, *p* = .001), had experience in PCA education (*β* = .26, *p* = .002), or those that work in a surgical ward with between 5 and 10 years of experience (*β* = .23, *p* = .009) had high knowledge and attitude scores for PCA, and the explanatory power was 26.1% (F = 7.83, *p* < .001) (Table 5).

**Table 5:** Factors affecting nurses’ knowledge and attitudes toward PCA

Variables	B	SE	*β*	t	*p*	95% CI
Constant	53.85	1.09	–	49.49	<.001	51.70–56.00
≥Postgraduate	1.35	1.73	.08	0.78	.438	−2.08–4.77
Experience of education on PCA	3.51	1.11	.26	3.18	.002	1.32–5.70
Clinical surgical ward experience (5–10 yr)	3.35	1.26	.23	2.66	.009	0.85–5.85
Clinical surgical ward experience (≥10 yr)	3.64	1.50	.24	2.43	.017	0.67–6.61
Ward (Upper abdomen)	3.73	1.08	.30	3.45	.001	1.59–5.88
Ward (Lower abdomen)	2.38	1.09	.19	2.19	.030	0.23–4.53
	R^2^ = 0.299, Adj. R^2^ = 0.261, F = 7.83, *p* < .001 Durbin-Watson = 2.08, Tolerance = 0.64–0.95, VIF = 1.05–1.55					

(*N* = 117)

*SE* standard error; *CI* confidence interval; *PCA* patient-controlled analgesia; *VIF* Variation Inflation Factor

## Discussion

This study was a cross-sectional study conducted to confirm the knowledge and attitude of surgical ward nurses toward PCA to develop future PCA education programs for nurses. Our findings confirmed that the total score of nurses’ knowledge and attitudes was not high. Although the tool used in this study has not been previously used, the knowledge score was similar to previous studies [23, 25]. Knowledge and attitude scores, according to the general characteristics of nurses, were also similar to those found in previous studies [15–17, 23]. Previous studies [15–17, 23] confirmed that knowledge was greater, or attitudes were more appropriate, among nurses receiving pain management education, and similar results were confirmed in this study. However, in this study, 80% of nurses had previously received pain management education, including education on PCA. To increase the effectiveness of education programs, it is important to understand factors such as the clinical experience, and educational background, and insufficient knowledge areas of individual nurses. This study suggests that intensive education should target newly trained nurses, who have little clinical experience on the surgical ward and who have received no previous education on PCA.

### Nurses’ knowledge of PCA

In this study, knowledge of opioid analgesics was relatively poorer than that of other areas, ranking second to fifth according to the overall incorrect answer rate (Table 2). However, awareness of addiction and respiratory depression, which have been identified as obstacles to sufficient postoperative pain control, was higher than that found in previous studies [1, 3, 6, 10, 19, 24]. Importantly, the question “If delirium occurs, PCA should be stopped” scored a high number of incorrect answers. Delirium is challenging in the postoperative recovery process, and nurses should be attentive to the use of opioid analgesics, particularly among the elderly. Postoperative pain may increase the occurrence of delirium, so PCA has been recommended [26, 27], although most nurses in this study were not aware of this recommendation. Additionally, among the drugs used in PCA, fentanyl citrate is known to be the safest drug for patients with impaired kidney function because it does not produce active metabolites that can cause side effects on the central nervous system [2]. However, only half of the nurses were aware of this. Previous studies [5, 20], demonstrated that PCA was a safer and more effective analgesic method than PRN because dosing can compensate for differences between individuals regarding drug requirements, but about half of the nurses believed the opposite. Previous studies have shown that nurses’ knowledge of analgesics, particularly opioid analgesics, is limited [17–19, 25]. Similarly, our study found that the overall knowledge about opioid analgesics used in PCA among nurses in the surgical ward was insufficient. Therefore, the future PCA education programs for nurses should be followed by a basic understanding of opioid analgesics. It is necessary to check whether the knowledge of nurses has improved by the effect of education. Accordingly, it is also necessary to identify whether the patient’s pain control is appropriate, and the patient’s satisfaction is increased.

### Nurses’ attitudes toward PCA

Like knowledge responses, the highest attitude scores were in the management domain, while inappropriate attitudes were observed in the opioid analgesic domain (Table 3). PCA opioid analgesics are not the only cause of nausea and vomiting occurring after surgery. However, in this study, over 50% of surgical ward nurses tended to stop PCA immediately when side effects manifested themselves. Nausea and vomiting are common postoperative complications caused by various factors including postoperative pain [28]. Moreover, according to previous studies [5, 20], PRN administration showed no difference in the incidence of side effects, such as nausea and vomiting, compared with PCA use, and patients using PCA had good pain control, high satisfaction, and shorter hospital stays. Therefore, if the patient’s pain intensity is continuously severe, PCA should not be stopped only because patients exhibit symptoms of nausea and vomiting. Rather, nurses should be educated on how to simultaneously improve nausea, vomiting, and pain by combining PCA and antiemetic drugs. Therefore, when developing a PCA education program, the educational content should be organized so that it combines the side effects of opioid analgesics with the basic understanding of causes, risk factors, and management of postoperative nausea and vomiting.

As shown in Tables 2 and 3, most nurses provide PCA education immediately after surgery, and this finding was consistent with that of previous studies [23]. However, it may be difficult for patients to accurately understand PCA immediately after surgery. Previous studies have confirmed that receiving this information before surgery has a higher educational effect [4, 8, 21]. Furthermore, Lin et al. [21] reported that patients educated about PCA before surgery, had a lower number of requests for help to the PCA nurse, which in turn increased work satisfaction for nurses. Therefore, it may be necessary to modify the policy of the entire hospital to increase both the satisfaction of nurses and patients when using PCA. It would be more effective to modify PCA policies regarding preoperative patient education at the hospital level rather than at the ward level or at the discretion if individual nurses.

### Correlation between knowledge and attitude subdomains on PCA

In this study, two knowledge and three attitude subdomains showed significant correlations with one another (Table 4). Specifically, the greater the knowledge about opioid analgesics, the better the attitude toward opioid side effect management. Previous studies have reported that doctors’ and nurses’ knowledge and attitudes are also important in determining the effectiveness and safety of PCA [6]. Furthermore, King and Walsh [24] reported that nurses’ fears of the effects and side effects of opioid analgesics disappeared after receiving education on PCA, and PCA education for patients became more effective. In this study, patients were also asked about their intentions regarding PCA education, and 95.7% of nurses answered that they required further education. Among the six domains investigated, the need for managing the side effects of opioid analgesics was the highest (66.7%), followed by basic knowledge education on opioid analgesics (47.9%). And nurses preferred a shortened PCA education program time of less than 30 minutes (53.8%). Therefore, the education program should consist of different domains including basic knowledge, opioid side effects, and the management of side effects. Role-playing exercises and case studies should also be actively utilized because they can confirm the status of nurses’ knowledge and evaluate whether the nurses demonstrate appropriate attitudes toward patient pain management.

### Factors affecting nurses’ knowledge and attitudes toward PCA

As set out in Tables 1 and 5, there was an association between the type of ward in which the nurse worked, and the knowledge acquired by nurses. Low knowledge scores were found among nurses on the musculoskeletal surgical ward and there was also a higher percentage of persons discontinuing PCA [14]. Since musculoskeletal surgical ward patients take oral painkillers earlier than patients on abdominal surgical wards, the use of PCA may be affected. Nevertheless, the reasons underlying the differences among nurses are uncertain. It is important to first identify these reasons and then provide targeted intensive education where required. It would also be useful to develop an educational program that compares the causes of different educational results after nurses receive PCA education in pairs between ward nurses who had a positive approach to nursing education and nurses who did not. Through this approach, nurses can independently identify the causes of the different educational results and help each other to increase their knowledge.

### Limitations and meanings

From a total of 22 surgical wards of tertiary hospital A, 8 surgical wards were selected in this study and sampled using the cluster sampling method. Since this study did not apply to all surgical ward nurses at a tertiary hospital, it is difficult to generalize the results to all surgical ward nurses. Furthermore, the knowledge and attitude scores may be lower in hospitals that are smaller than those included in this study and where pain management education including PCA is not regularly conducted. This cross-sectional study has all the limitations and risks of bias inherent to cross-sectional studies. Specifically, as participants of various ages with different empirical backgrounds were sampled at the same time, the effect of exogenous variables, such as clinical experience, cannot be reduced, and this sample cannot represent all age groups. We consider that further studies should verify the validity and reliability of the tools developed and used in this study.

Nevertheless, the results of this study can be considered meaningful. Most previous studies have measured the overall knowledge and attitude of nurses toward pain management. There are few studies confirming nurses’ knowledge and attitudes toward PCA, and these did not specifically identify the weak and strong parts of PCA education. The strength of this study was that it identified which aspects of nurses’ knowledge and attitudes were vulnerable and what types of intensive education are necessary when educating about PCA.

## Conclusions

This study sought to identify trends in the educational direction of PCA that will be helpful to nurses in the future by confirming their knowledge and attitudes toward PCA. Our study showed that the average knowledge and attitude toward PCA scores of surgical ward nurses were not high. Nevertheless, our results confirmed that nurses with extensive clinical experience on a surgical ward and those who received education on PCA, had better attitudes toward PCA. Furthermore, greater the knowledge of opioid analgesics was associated with better the attitude toward managing the side effects of opioid analgesics. This study suggests a number of approaches to increase the educational effect of PCA. First, as a hospital policy, the timing of the preoperative delivery of PCA information to patients should be standardized. In addition, in-hospital programs should be developed for newly trained nurses working on surgical wards to educate them on basic knowledge, side effects, and management of the side effects of opioid analgesics. Finally, a hospital’s support policy will be required to conduct follow-up studies on whether nurses’ knowledge and attitudes change after receiving education on PCA. These follow-up studies can confirm that the effectiveness of the revised PCA education program has improved nurse knowledge levels and attitudes, and track whether these changes lead to improvements in patient pain management outcomes.

## Data Availability

All data generated or analyzed during this study are included in Tables 1 to 5. The other raw datasets generated during the current study and not included in this published article are not available because ethical approval and participant consent prohibit public exposure. However, the datasets used and/or analyzed during the current study are available from the corresponding author on reasonable request.

## Abbreviations

ANOVA: Analysis of variance
APS: Acute pain service
PCA: Patient-controlled analgesia
PRN: Pro re nata
